# Dendritic cells infected with recombinant adenoviral vector encoding mouse fibroblast activation protein‐α and human livin α exert an antitumor effect against Lewis lung carcinoma in mice

**DOI:** 10.1002/iid3.1011

**Published:** 2023-09-27

**Authors:** Zaiting Ye, Jiongwei Pan, Zhangyong Yin, Shuanghu Wang, Yuling Li, Xiaoping Cai, Hao Zheng, Zhuo Cao

**Affiliations:** ^1^ Department of Radiology The Sixth Affiliated Hospital of Wenzhou Medical University Lishui Zhejiang China; ^2^ Department of Respiratory The Sixth Affiliated Hospital of Wenzhou Medical University Lishui Zhejiang China; ^3^ Department of Medicine Lishui People's Hospital Lishui Zhejiang China

**Keywords:** antitumor immunity, cancer‐associated fibroblasts, fibroblast activation protein‐α, Lewis lung carcinoma, livin α

## Abstract

**Background:**

Fibroblast activation protein‐α (FAP) and livin α are considered as cancer‐associated fibroblasts (CAFs) and tumor‐specific targets, respectively, for immunogenic tumor vaccines. This study is designed to decipher the antitumor effect of double‐gene modified dendritic cells (DCs) on Lewis lung carcinoma (LLC).

**Methods:**

By encoding mouse FAP cDNA and human livin α (i.e., hlivin α) cDNA into recombinant adenoviral vector (rAd), rAd‐FAP, rAd‐hlivin α, and rAd‐FAP/hlivin α were constructed, which were then transduced into mouse DCs. LLC‐bearinig mice were immunized with the infected DCs (5 × 10^5^ cells/mouse), followed by calculation of tumor volume and survival rate. The identification of CAFs from mouse LLC as well as the determination on expressions of FAP and livin α, was accomplished by western blot. Cytotoxic T lymphocyte assay was harnessed to assess the effect of the infected DCs on inducing splenic lymphocytes to lyse CAFs.

**Results:**

DCs were successfully transduced with rAd‐FAP/hlivin α in vitro. FAP was highly expressed in CAFs. CAFs were positive for α‐SMA and negative for CD45 and CD31. Livin α level was upregulated in mouse LLC. Immunization with rAd‐FAP/hlivin α‐transduced DCs suppressed LLC volume and improved the survival of tumor‐bearing mice. Immunization with rAd‐FAP/hlivin α‐transduced DCs enhanced the cytotoxic effect of splenic lymphocytes on LLC tumor‐derived CAFs.

**Conclusion:**

Injection with rAd‐FAP/hlivin α‐transduced DCs promotes immune‐enhanced tumor microenvironment by decreasing CAFs and suppresses tumor growth in LLC mouse models.

## 1 INTRODUCTION

Lung cancer is the second most common type of malignancy in the world, with which patients generally have a low 5‐year survival rate and poor prognosis.^1^ Although surgery, radiotherapy, chemotherapy, and targeted therapy comprise the general treatment for patients with lung cancer, the effectiveness of treatment is limited by the stage of disease, heterogeneity, severe side effects, and the genetic profile of the tumor.^2^, ^3^, ^4^, ^5^

With the advance of biological therapy, there is a growing interest in the tumor‐specific cytotoxicity of cytotoxic T lymphocytes (CTLs).^6^ Previously, increasing the number of CTLs and activating CTLs towards effector cells against cancer cells have been considered promising strategies for making efficient antitumor immune responses.^7^ It is believed that CTLs with CD8^+^ surface marker are primed by dendritic cells (DCs), CD4^+^ T cells, and natural killer cells.^8^ Among them, DCs play a central role in the immune response and are able to induce tumor‐specific CTL response by professionally presenting antigens to naïve T lymphocytes, thereby activating CTL to destroy antigen‐expressing cancer cells.^9^ However, cancer cells and stromal cells within the tumor microenvironment (TME) exert immunosuppressive properties that disturb the antitumor immunity of DCs.^10^ Therefore, it is important to genetically modify DCs based on appropriate tumor targets for the development of effective tumor vaccines.

Fibroblast activation protein‐α (FAP) is a membrane‐bound serine protease and is specifically expressed on the surface of reactive cancer‐associated fibroblasts (CAFs) that constitute a major stromal component of most solid tumors.^11^ Available evidence has demonstrated that FAP plays a critical role in the formation of TME, in which this cell surface protease can shape key features of CAFs through proteome and degradome alterations to suppress antitumor immunity and facilitate tumor growth and metastasis.^12^ In recent years, the depletion of FAP in CAFs by multiple methods, including cell‐based vaccines has been reported to induce the immune system and inhibit tumor progression.^13^, ^14^

Livin belongs to the family of antiapoptotic proteins and is poorly expressed in most normal tissues but overexpressed in most common human cancer cells.^15^, ^16^ In view of the contributing role of livin in proliferation, antiapoptosis, and resistance of cancer cells as well as good prognosis,^17^, ^18^, ^19^ it has been widely thought as a specific gene in tumor tissues and could be used as a promising target for tumor immunotherapy.

Previously, we confirmed that human livin α (i.e., hlivin α)‐transduced DCs enhanced tumor‐specific CTL response for killing Lewis lung carcinoma (LLC) cells as well as inhibited tumor growth in mice, yet this antitumor vaccination failed to realize tumor eradication, as the tumor‐bearing mice eventually died from the tumors (Junping^20^). In this study, we modified mouse bone marrow‐derived DCs by infecting the cells with recombinant adenoviral vector (rAd) encoding transgenes to explore the immunogenicity of mouse FAP combined with human livin α on LLC in mice so as to improve the efficacy of LLC vaccine.

## 2 METHODS

### Experimental animals and ethics statement

Female C57BL/6 (H‐2^b^) mice (6–8 weeks old) were reared in a pathogen‐free environment with food and drinking water ad libitum. All experiments involving animals in this study had been granted by the Ethics Committee of Zhejiang Baiyue Biotech Co., Ltd. for Experimental Animals Welfare (Approval No. ZJBYLA‐IACUC‐20221104), and all procedures abided by the guidelines of the China Council on Animal Care and Use.

### 2.1 Cell line and culture

LLC cells with H‐2^b^ background (iCell‐m027) were purchased from iCell Bioscience and cultured in Dulbecco's modified eagle medium (DMEM; 10566016, Thermo Fisher) containing 10% fetal bovine serum (FBS; 10100147, Thermo Fisher) and 1% penicillin‐streptomycin solution (G4003, Servicebio). HEK‐293 cells (CL‐0001, Procell) were maintained in minimum essential medium (PM150467, Procell) supplemented with 10% FBS and 1% penicillin‐streptomycin solution. Cell culture was carried out in a 5% CO_2_ incubator at 37°C.

### 2.2 Construction of rAd

Using pIRES vector (VT1058, Youbio) as the backbone, FAP full‐length cDNA (NM_007988.3) was inserted between EcoRⅠ and BamHⅠ cleavage sites to construct the pIRES‐FAP plasmid. The forward polymerase chain reaction (PCR) primer was 5′‐GCTAGCATGAAGACATGGCT‐3′. The reverse primer was 5′‐ACGCGTTCAGTCTGATAAAGAA‐3′. The construction of human livin α cDNA plasmid was conducted as previously described (Junping^20^). The forward primer was 5′‐GACCACGTGGATGGGCAGAT‐3′, and the reverse primer was 5′‐TTGCACGTCCTCTCCTCCTG‐3′. Livin α cDNA from pIRES2‐EGFP‐human (h) livin α plasmid (Dr. Riki Perlman, Department of Hematology, Hadassah‐Hebrew University Medical Center, Jerusalem, Israel) was amplified with specific primers by PCR. Then, the PCR products were inserted into the multiple cloning site B of pIRES‐FAP plasmid to obtain pIRES‐FAP/hlivin α plasmid. Next, shuttle plasmid was generated by inserting the amplified cDNA from pIRES‐FAP, pIRES2‐EGFP‐hlivin α or pIRES‐FAP/hlivin α into pDC316‐mCMV‐EGFP vector (HG‐VXY0585, HonorGene), and then cotransfected with the helpler plasmid [pBHGlox(delta)E1,3Cre] into HEK‐293 cells under the help of Lipofectamine 2000 reagent (11668027, Thermo Fisher). The construction of rAd‐containing mouse FAP or/and human livin α cDNAs (rAd‐FAP, rAd‐hlivin α, rAd‐FAP/hlivin α) was subsequently performed using AdMax Kit D system (Microbix Biosystems Inc.). After amplification, the purified virus was determined by endpoint dilution assay (at cell passage 4) and PCR (at cell passage 2). Later, the negative control rAd‐containing EGFP (rAd‐EGFP) was generated following the same procedures.

### 2.3 Culture and infection of dendritic cells (DCs)

DCs were harvested from mouse bone marrow as per previous guidance,^14^ and cultured in 6‐well plates (1 × 10^6^ cells/well) with complete RPMI‐1640 medium (PM150110B, Procell) supplemented with 20 ng/mL recombinant murine granulocyte–macrophage colony‐stimulating factor (GM‐CSF; SRP3201, Sigma‐Aldrich) and 20 ng/mL recombinant murine interleukin‐4 (IL‐4; M10465, Abmole) at 37°C with 5% CO_2_. The non‐adherent cells were collected with 0.5 mL serum‐free medium containing GM‐CSF (20 ng/mL) and IL‐4 (10 ng/mL) on Day 7 and transferred in 24‐well plates (5 × 10^5^ cells/well). Next, cell infection with rAd‐EGFP, rAd‐FAP, rAd‐hlivin α, or rAd‐FAP/hlivin α at a multiplicity of infection of 200 was performed for 2 h, followed by an additional 48‐h incubation. Afterwards, the infected DCs were washed with phosphate‐buffered saline (PBS; G4202, Servicebio) and used for in vitro and in vivo experiments.

### 2.4 Establishment of LLC mouse models and in vivo immunization

The cultured LLC cells were digested and collected in PBS at a concentration of 2.5 × 10^6^ cells/mL. To establish LLC‐bearing mice, 5 × 10^5^ cells were subcutaneously injected into the right flank of each mouse.^14^

To investigate the antitumor activity of DC vaccine in vivo, LLC mouse models, whose tumor diameter reached 4–6 mm on the 8th day after LLC cell injection, were randomly divided into four groups (*n* = 10), and mice in each group were subjected to immunization with rAd‐EGFP‐transduced DCs (1#), rAd‐hlivin α‐transduced DCs (2#), rAd‐FAP‐transduced DCs (3#), and rAd‐FAP/hlivin α‐transduced DCs (4#) (5 × 10^5^ cells/mouse), respectively. Immunization was given by subcutaneous injection into the left flanks of mice for a total of three times every 3 days. Every 2 days after the first vaccination, the longest diameter (length) and shortest diameter (width) of mouse tumors were measured with a vernier caliper to calculate the tumor volume following the formula: *V* = length × width^2^ × 0.52 (mm^3^) (Junping^20^). All mice were euthanized (50 mg/kg pentobarbital sodium, P‐010, Sigma‐Aldrich) when the tumor diameter reached 20 mm in vivo, with death time recorded accordingly for calculation of survival rate. The observation period for the survival rate of tumor‐bearing mice lasted for 80 days.

### 2.5 CAF isolation

The isolation of CAFs from implanted LLC was conducted as described previously.^21^ In brief, tumor tissues were excised from mice about 18 days after LLC cell injection and cut into 1–2 mm^3^ pieces. 10% FBS‐containing DMEM was used to cover tissue pieces in T25 cell culture flasks at 37°C with 5% CO_2_, with medium replaced every 48 h. After 2 weeks, trypsinization (0.25% trypsase, C0201, Beyotime) was performed for 5 min, and cell pellets were allowed to grow adherently in the medium, followed by PBS washing. The purified CAFs were normally cultured and used for following assays at Passage 4–5.

### 2.6 Western blot

Tumor tissues, CAFs or DCs were homogenized in RIPA buffer (R0010, Solarbio) at 4°C to lyse total protein, followed by quantification of protein concentration using BCA Protein Assay Kit (23225, Thermo Fisher). Equal amount of protein samples was electrophoresed by 10% sodium dodecyl sulfate‐polyacrylamide gel electrophoresis and transferred to polyvinylidene fluoride membranes (IPVH08100, Sigma‐Aldrich). Following incubation with blocking buffer (P0231, Beyotime), Western blot was performed by incubating the membranes with primary antibodies against FAP (NB110‐85534, 88 kDa, Novus Biologicals), livin α (NB100‐56145, 33 kDa, Novus Biologicals), CD45 (ab10558, 147 kDa, Abcam), CD31 (ab281583, 82 kDa, Abcam), α‐SMA (ab5694, 42 kDa, Abcam), and internal control GAPDH (ab8245, 37 kDa, Abcam) at 4°C overnight. The next day, horseradish peroxidase‐conjugated secondary antibodies (ab205718, ab6728, Abcam) were utilized to incubate the membranes at room temperature for 2 h. Immunoblots were revealed using ECL luminescence reagent (C510043, Sangon Biotech), and relative protein expressions were analyzed by Image Quant LAS 4000 system (GE Healthcare).

### 2.7 CTL assay

Splenic lymphocytes were obtained from mice on Day 7 after receiving the last dose of DCs vaccination and then were cultured in complete medium (CM‐M153, Procell) at 37°C with 5% CO_2_ for 90 min. CAFs pretreated with Mitomycin C (25 mg/L, M5791, Abmole) were used to stimulate splenic lymphocytes in the medium containing IL‐2 (M19999, Abmole). After 5 days of incubation, the stimulated lymphocytes were harvested as effector cells and then added into 96‐well plates with CAFs (target cells) at a ratio of 20:1, 40:1, or 60:1. The cytotoxic effect of effector cells on target cells was examined by Cytotox96 Non‐Radioactive Cytotoxicity Assay Kit (G1780, Promega) according to the manufacturer's protocol. A microplate reader (Multiskan FC, Thermo Fisher) was employed to detect cell absorbance at 450 nm, followed by cytotoxicity calculation.^22^

### 2.8 Statistical analysis

Measurement data were obtainable from three repeated experiments and shown as mean ± standard deviation. Comparisons among different groups were analyzed using one‐way analysis of variance, and comparison between two groups in Figure 2 was analyzed using independent samples *t*‐test. Survival was analyzed by Kaplan–Meier survival curve and log‐rank test. GraphPad Prism 8.0 (GraphPad Software Inc.) was utilized for statistical analysis, and statistical significance was set at *p* < .05.

## 3 RESULTS

### 3.1 DCs were successfully transduced with rAd‐FAP/hlivin α in vitro

**Figure 1:**
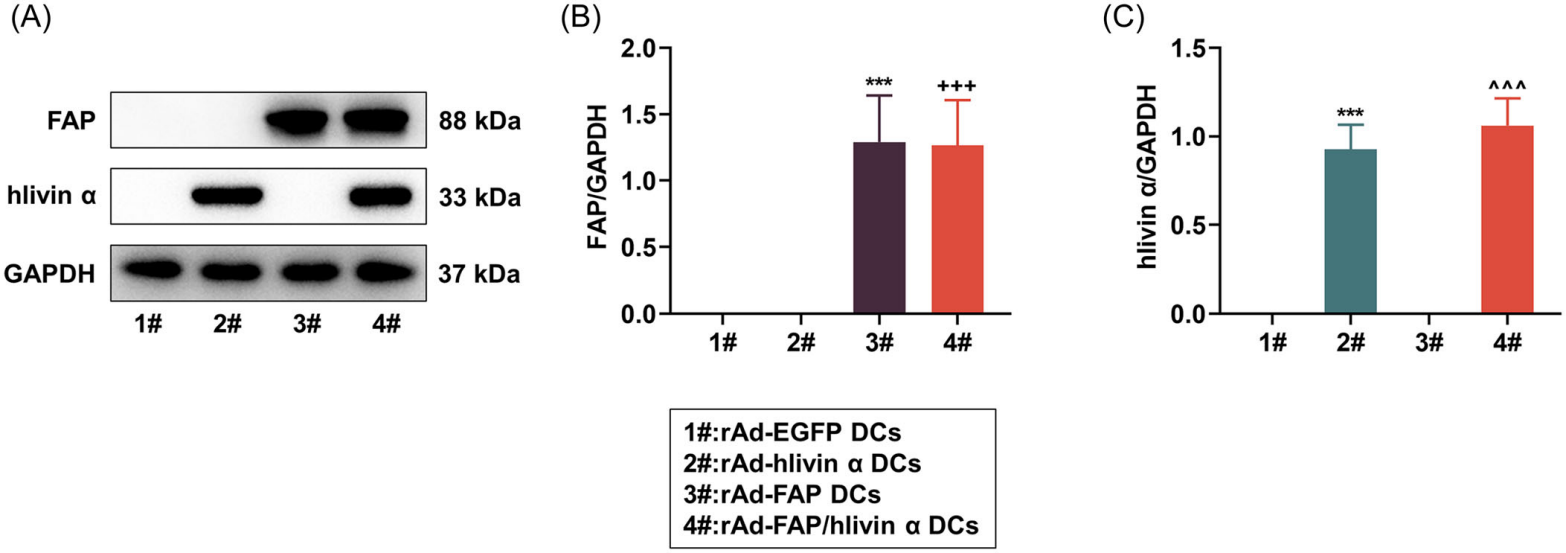
Identification of rAd‐infected DCs in vitro. (A–C) rAd‐FAP, rAd‐hlivin α, and rAd‐FAP/hlivin α were constructed using mouse FAP cDNA and human livin α cDNA, and then transduced into mouse bone marrow‐derived DCs, respectively. For evaluation of transduction efficacy, western blot was performed to measure protein expressions of FAP and livin α in cells, with GAPDH functioned as a loading control. ***p < .001, versus 1#; ^+++^ *p* < .001, versus 2#; ^^^^^ *p* < .001, versus 3#. DCs, dendritic cells; FAP, fibroblast activation protein‐α; rAd‐FAP, recombinant adenoviral vector encoding mouse FAP cDNA; rAd‐hlivin α, recombinant adenoviral vector encoding human livin α cDNA.

After mouse DCs were infected with rAd carrying FAP or/and hlivin α, western blot was performed to analyze transduction efficiency. Compared with that in rAd‐EGFP‐infected cells, an increasing trend towards the expression of FAP was observed in rAd‐FAP‐infected cells, and an elevated tendency towards the expression of livin α was viewed in rAd‐hlivin α‐infected cells (Figure 1A–C, *p* < .001). In rAd‐FAP/hlivin α‐infected cells, FAP and livin α expressions were both increased when compared with those in rAd‐EGFP‐infected cells and rAd‐hlivin α‐infected cells (Figure 1A–C, *p* < .001). Collectively, these findings indicated the successful transduction of DCs with rAd‐FAP and rAd‐livin α in vitro.

### 3.2 FAP was highly expressed in CAFs from mouse LLC

**Figure 2:**
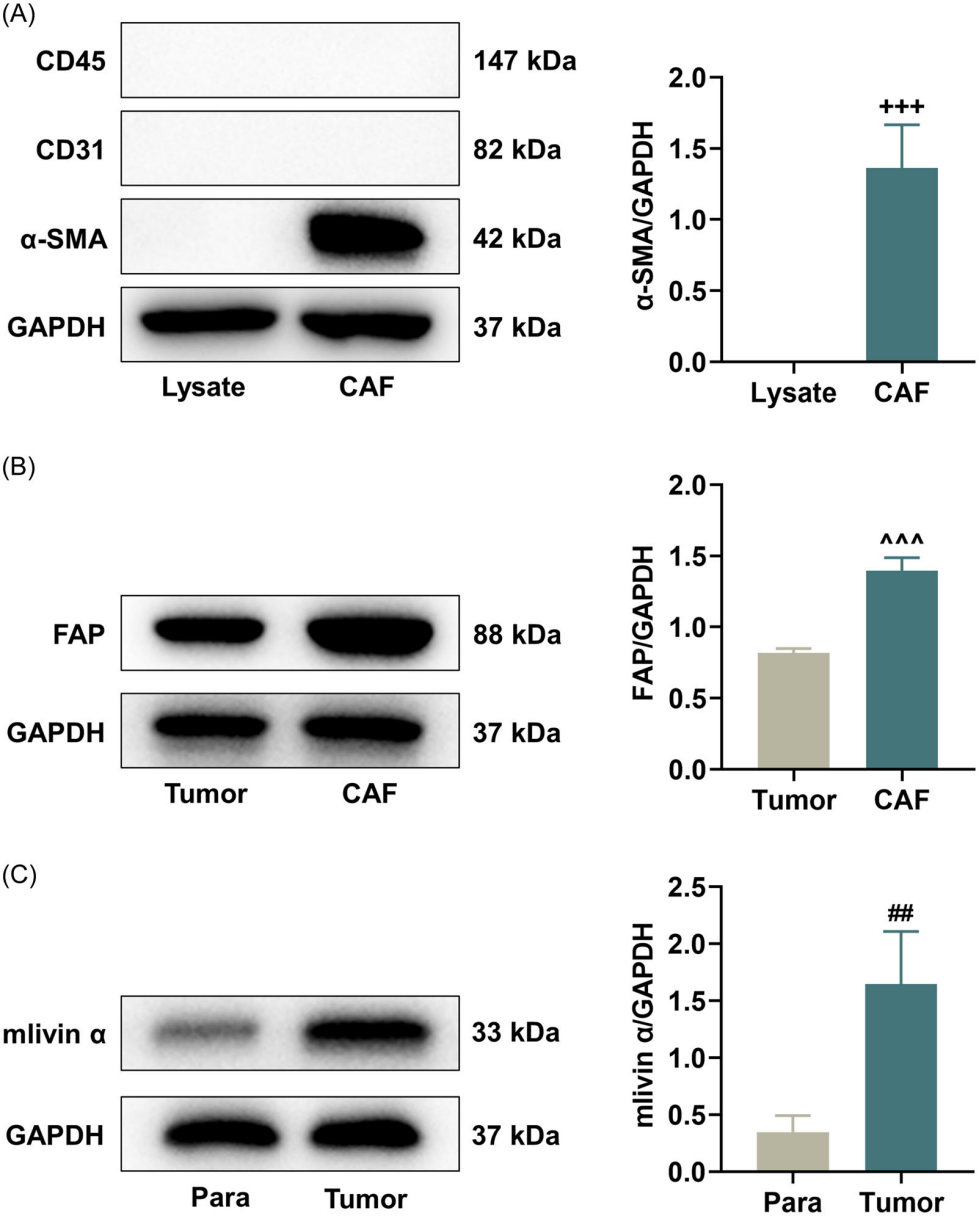
Identification of LLC tumor‐isolated CAFs and determination on expressions of FAP and livin α in LLC. (A–C) Western blot was performed to measure protein expressions of CD45, CD31, α‐SMA, and FAP in CAFs isolated from LLC‐bearing mice as well as the protein expression of livin α in mouse LLC tumors. GAPDH was used as the loading control. ^+++^ *p* < .001, versus lysate; ^^^^^ *p* < .001, versus tumor; ^##^ *p* < .001, versus Para. CAFs, cancer‐associated fibroblasts; LLC, Lewis lung carcinoma; Para, paracancerous tissues.

Next, we isolated CAFs from LLC tumor‐bearing mice to detect epithelial cell adhesion molecules (CD45 and CD31) and α‐SMA. The results of western blot verified that CAFs were positive for α‐SMA protein (Figure 2A, *p* < .001) and negative for CD45 and CD31 proteins. Compared with that in mouse LLC, FAP level was found to be significantly upregulated in CAFs (Figure 2B, *p* < .001).

### 3.3 Livin α level was upregulated in mouse LLC

It has previously confirmed that livin α is specifically expressed in multiple tumor cells, and its downregulation shows an antitumor effect.^23^ Moreover, the expression of livin α was demonstrated to be increased in mouse LLC tissues, as compared with that in paracancerous tissues (Figure 2C, *p* < .01).

### 3.4 Immunization with rAd‐FAP/hlivin α‐transduced DCs suppressed LLC volume and improved the survival of tumor‐bearing mice

**Figure 3:**
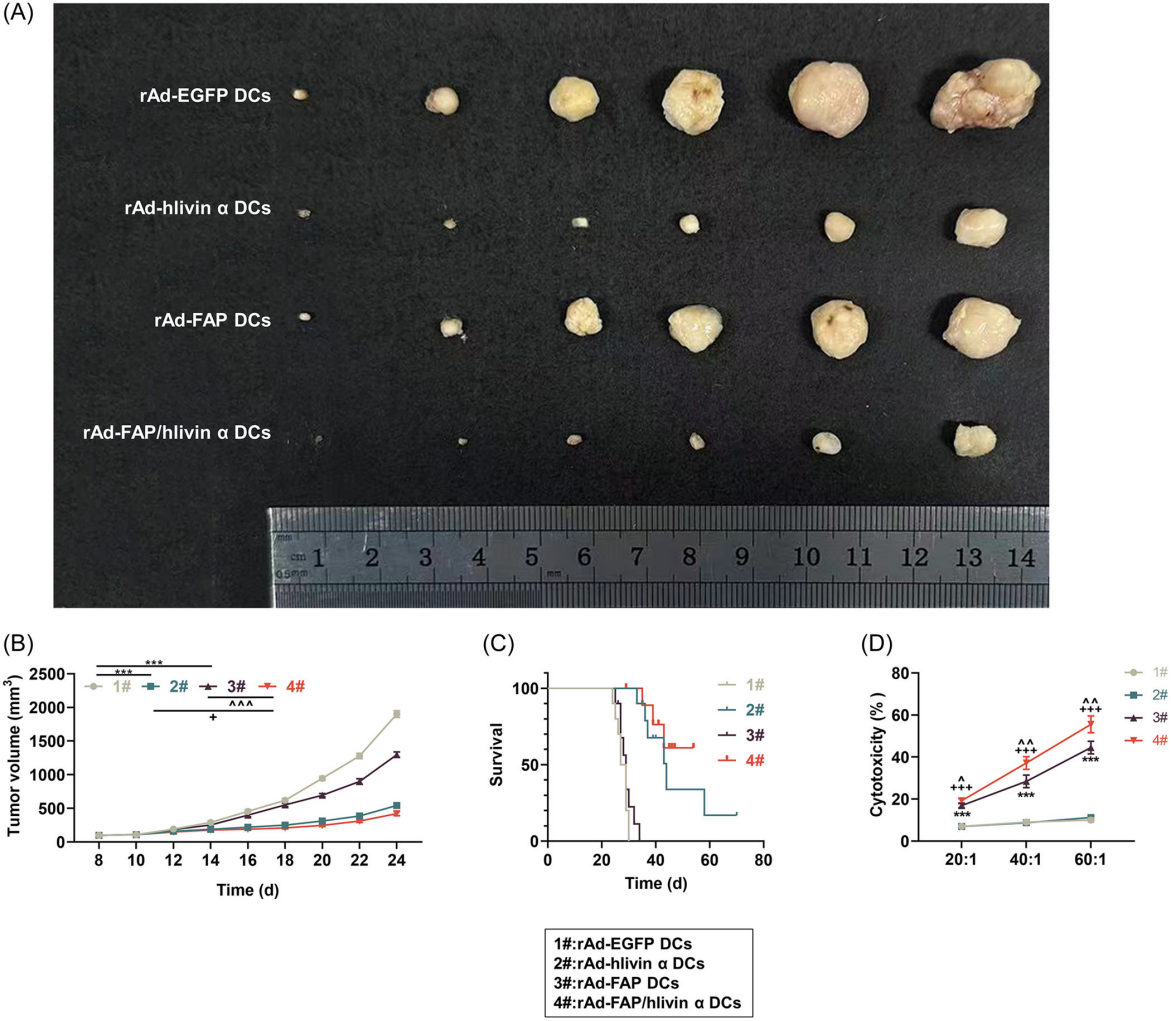
The antitumor effect of rAd‐FAP/hlivin α‐transduced DCs on LLC in mice. (A and B) Mice were separately immunized with rAd‐EGFP‐transduced DCs, rAd‐FAP‐transduced DCs, rAd‐hlivin α‐transduced DCs, and rAd‐FAP/hlivin α‐transduced DCs (5 × 10^5^ cells/mouse) on Day 8 postinjection with LLC cells, and tumor volume (mm^3^) was calculated every 2 days for a total 24 days. (C) Mouse survival rate was observed for 80 days. (D) Splenic lymphocytes were obtained from mice on Day 7 after receiving injection with rAd‐EGFP‐transduced DCs, rAd‐FAP‐transduced DCs, rAd‐hlivin α‐transduced DCs or rAd‐FAP/hlivin α‐transduced DCs. The cytotoxic effect of splenic lymphocytes on LLC‐derived CAFs was analyzed using Cytotox96 Non‐Radioactive Cytotoxicity Assay Kit. ***p < .001, versus 1#; ^+^ *p* < .05, ^+++^ *p* < .001, versus 2#; ^^^ *p* < .05, ^^^^ *p* < .01, ^^^^^ *p* < .001, versus 3#. CAFs, cancer‐associated fibroblasts; DCs, dendritic cells; LLC, Lewis lung carcinoma; FAP, fibroblast activation protein‐α; rAd‐FAP, recombinant adenoviral vector encoding mouse FAP cDNA; rAd‐hlivin α, recombinant adenoviral vector encoding human livin α cDNA.

In the following therapeutic efficacy study, the volume of LLC in mice injected either with rAd‐hlivin α‐transduced DCs or rAd‐FAP‐transduced DCs was decreased (Figure 3A, *p* < .001), and this suppressing effect was strengthened in the presence of rAd‐FAP/hlivin α‐transduced DCs (Figure 3A,B, *p* < .05). Moreover, tumor‐bearing mice who received immunization with rAd‐FAP/hlivin α‐transduced DCs showed higher survival rate compared with those receiving immunization with rAd‐hlivin α‐transduced DCs or rAd‐FAP‐transduced DCs alone (Figure 3C, Table 1).

**Table 1:** Statistical parameters of Kaplan–Meier survival curve results.

*T* a	*d* b	*n* ^c^	1 − *d*/*n*	S (*t*)^d^
1#				
0	‐	10	‐	1.000
24	1	9	0.889	0.900
25	1	8	0.875	0.800
26	1	7	0.857	0.700
27	2	5	0.600	0.500
29	2	3	0.333	0.300
30	2	0	‐	0.000
2#				
0	‐	10	‐	1.000
33	1	9	0.889	0.900
36	1	7	0.857	0.700
37	1	6	0.833	0.600
43	1	3	0.667	0.300
44	1	2	0.500	0.200
58	1	1	0.000	0.100
3#				
0	‐	10	‐	1.000
25	1	9	0.889	0.900
27	2	6	0.667	0.600
28	1	5	0.800	0.500
29	2	3	0.333	0.300
30	1	2	0.500	0.200
32	1	1	0.000	0.000
34	1	0	‐	0.000
4#				
0	‐	10	‐	1.000
35	1	9	0.889	0.900
39	1	8	0.875	0.800
43	1	4	0.750	0.400

^a^ *T*, survival time.

^b^ *d*, the number of event occurrences.

^c^ *n*, the number of an individual still alive at time T.

^e^ *S* (*t*), the probability of an individual surviving beyond time T.

### 3.5 Immunization with rAd‐FAP/hlivin α‐transduced DCs enhanced the cytotoxic effect of splenic lymphocytes on LLC‐derived CAFs

With regard to the contributing role of CAFs in the formation of tumor microenvironment, we investigated whether rAd‐FAP/hlivin α‐transduced DCs could enhance the cytotoxic effect of splenic lymphocytes on CAFs in vitro. As shown in Figure 3D, splenic lymphocytes from mice immunized with rAd‐FAP‐transduced DCs exhibited an increased cytotoxicity to CAFs (*p* < .001), whereas splenic lymphocytes from mice immunized with rAd‐hlivin α‐transduced DCs showed no significant cytotoxic effect on CAF. Of note, splenic lymphocytes from mice immunized with rAd‐FAP/hlivin α‐transduced DCs exerted stronger cytotoxicity against CAFs than those from mice immunized with rAd‐hlivin α‐transduced DCs or rAd‐FAP‐transduced DCs alone (Figure 3D, *p* < .05).

## 4 DISCUSSION

Although targeted therapy and immune checkpoint inhibitors have improved the treatment landscape for patients with lung cancer in recent years, the clinical efficacy is still unsatisfactory.^24^ Hence, the tremendous endeavor has been dedicated to finding novel and effective therapy with low toxicity. In this study, two‐antigen‐loaded DC vaccines are successfully built through infection with rAd encoding mouse FAP and human livin α, which have been reported as an efficient tool for transferring genes into DCs.^19^ Based on the results of the therapeutic efficacy study in vivo, we revealed for the first time that rAd‐FAP/hlivin α‐transduced DCs suppressed tumor volume and improved the survival of LLC mouse models, and these effects were more pronounced than those of rAd‐hlivin α‐transduced DCs or rAd‐FAP‐transduced DCs. Furthermore, we demonstrated that rAd‐FAP/hlivin α‐transduced DCs showed a more significant effect on inducing CTL responses to kill CAFs than rAd‐FAP DCs.

As the most potent antigen‐presenting cells, DCs have always been a major interest in the development of tumor vaccines.^9^, ^25^ Compared with antigen‐adjuvant vaccines, a previous study has suggested that antigen‐loaded DC vaccines exhibit a stronger effect on inducing T‐cell immune responses against mouse tumors.^26^ Recently, tumor‐associated antigen‐pulsed DC vaccines for cancer immunotherapy have been studied in clinical trials and reported to obtain a good therapeutic effect,^27^, ^28^ yet their effectiveness and sustained antitumor immunity remain controversial because of the adaptive immunomodulatory mechanisms of TME and the mutational nature of the cancer cell genome. Existing studies have documented that livin protein is overexpressed in many lung cancer cell lines and primary lung cancers and that many patients with lung cancer have anti‐livin antibodies, as well as anti‐livin cellular immune responses,^29^, ^30^, ^31^ hinting the potential of livin as a target for the immunotherapy of lung cancer. In this study, we also found that LLC excised from mice presented overexpression of livin α.

With an in‐depth understanding of tumorigenesis, researchers have discovered that CAFs are closely associated with the development of immunosuppression by interacting with immune cells in TME and have emerged as a novel interstitial target for tumor immunotherapies designed to complement cancer cell‐targeted therapies.^32^, ^33^ CAFs, which are characterized as α‐SMA‐marked myofibroblasts, show a more stable genome than cancer cells,^34^ suggesting that they have a low risk of antigen loss and treatment tolerance in the immunotherapy of solid tumors. It is widely known that FAP is highly expressed in tumor stroma from patients with lung cancer, and FAP overexpression can facilitate the proliferation of CAFs as well as cancer cells in vitro and in vivo.^35^ Compared with FAP‐negative CAFs, FAP‐positive CAFs have been found to facilitate tumor growth of gastric cancer in vivo as well as inhibit T‐cell activation and infiltration, which could be responsible for the failure of antitumor immunity.^36^ In the study of breast cancer, Xia et al. have indicated that FAP‐based vaccines can enhance the specific immune response for eliminating CAFs, which is an attractive way to overcome immunosuppression in combination with antitumor agents.^37^ In LLC mouse models, we confirmed that FAP was preferentially expressed in CAFs in comparison with that in solid tumors. Similar to the previous study,^14^ we observed a significant antitumor effect of rAd‐FAP‐transduced DCs on LLC by reducing tumor volume, increasing survival rate, and enhancing CAF‐specific cytotoxicity, and intriguingly this effect was further strengthened in the presence of livin α‐targeted vaccination. Taken together, it is indicated that double‐gene‐modified DC vaccines are more effective in combating LLC than single‐gene‐modified DC vaccines.

In conclusion, the present study suggests that injection with rAd‐FAP/hlivin α‐transduced DCs promotes immune‐enhanced TEM by killing CAFs and suppresses tumor growth, thereby prolonging mouse survival, which is an advancement in the field of tumor immunology. Also, our current findings support the advantage of DCs as efficient gene vehicles in the development of tumor vaccines. As FAP and livin α are specifically expressed in CAFs and LLC cells, respectively, immunotherapy based on the combination of the two may have a huge potential to overcome TEM‐caused immunosuppression and deliver a one‐two punch to LLC.

## AUTHOR CONTRIBUTIONS

**Zaiting Ye**: Conceptualization; writing—original draft; writing—review & editing. **Jiongwei Pan**: Conceptualization; writing—original draft; writing—review & editing. **Zhangyong Yin**: Data curation; formal analysis; resources; software. **Shuanghu Wang**: Investigation; supervision; validation. **Yuling Li**: Investigation; methodology; visualization. **Xiaoping Cai**: Software; supervision. **Hao Zheng**: Methodology; validation. **Zhuo Cao**: Project administration; supervision.

## CONFLICTS OF INTEREST STATEMENT

The authors declare no conflicts of interest.

## Data Availability

The analyzed data sets generated during the study are available from the corresponding author on reasonable request.
